# Alternate layer by layered self assembly of conjugated and unconjugated Salen based nanowires as capacitive pseudo supercapacitor

**DOI:** 10.1038/s41598-021-98288-y

**Published:** 2021-09-22

**Authors:** Mohammad Mahdi Doroodmand, Sina Owji

**Affiliations:** Department of Chemistry, College of Sciences, Shiraz University, 71454 Shiraz, Iran

**Keywords:** Chemistry, Analytical chemistry, Imaging studies, Nanoscience and technology, Nanoscale materials, Nanowires

## Abstract

A novel electrosynthetic method has been introduced based on alternate layer-by-layered self-assembly of conjugated/unconjugated Salen-based nanowires as a capacitive pseudo-supercapacitor. For this purpose, a three-electrode system consisted of a glassy carbon (*GC*), Ag/AgCl (*Sat’d Cl*^*−*^) and a Pt rod as working, reference, and counter electrodes, respectively. The electrolyte included the same molar concentration (0.040 mol L^−1^) of each Salen monomer (as initial precursor), and KCl solution (as supporting electrolyte), besides using KOH solution (0.01 mol L^−1^, as basic-controlling reagent) inside acetone/water (4:1, V/V) as a solvent. The formation of this self-assembly nanowire was attributed to the control of the electrical conductivity of this polymer during formation of an organometallic complex with K^+^ as responsible complex forming agent. This novel nanowire then played role as a capacitive pseudo-supercapacitor. Based on the chrono—potentiometry, reproducible charge/discharge process for at least 5000 cycles was observed at a potential between − 2.00 and + 1.75 V (vs. Ag/AgCl). The capacity behavior of the polymer was also evidenced using electrochemical impedance spectroscopy. This synthesized polymeric nanowire was adopted as the acceptable pseudo-supercapacitor with real capacity equals to 3110 ± 6 (n = 3) C g^−1^. This study was considered as the first report at which the self—assembly of organometallic compounds as an efficient pseudo—supercapacitor was introduced.

## Introduction

Besides the general metals and semiconductors, synthetic metals, due to the possessing significant characteristics such as controllable electrical conductivity, and well-behaved morphology, are considered as a good candidate in the future of the electronic systems^1^. These properties are often attributed to the controllable electrical conductivity of the conjugated π-bands all over some different molecules like the family of “*Schiff bases*” named as “*Salen*”^2–4^.

Compared to the introduced conductive polymers such as polyphenylene, polypyrenes and polypyrroles, the Salen-based polymers are considered as adequate organic conductor^2,3^. However, among these characteristics, self-assembly growth of these synthetic pseudo metals at three-dimensions often causes to play a role as an equivalent resistance element^4^. Conversely, precise adjusting the electrical resistivity of these equivalent elements is considered as the border awareness in the molecular electronic systems during the last decades^5^.

Among the introduced synthesis methods such as epoxidation of alkenes, oxidation of hydrocarbons and many other catalyzed reactions^6^, electrosynthesis is accepted as selective technique^4^. This technique often leads to synthesize structures with well-ordered morphology and adequate purity^4^. To operate the electrical resistivity of the conductive polymers such as Salen, frequently scientists are focused on the electro-oxidation process^3,4^, along with complexation with labile complexing agents such as Ni^2+^, Fe^3+^, Cu^2+^, etc.^3–6^.

About the metal/polymer complexes, this morphology limits the electrical conductivity to some special dimensions^6^. Consequently, the applications of these polymers have been restrained, owing to certain intrinsic challenges like low electrical conductivity^6,7^. The electrical resistivity of these metal/Salen complexing network is almost confined to the oxidation number of the metal ion(s), presented in the complex sphere^8^. These shortcomings clearly exhibit the strong demand for introducing new synthetic methods to control different features of the Salen-based polymers, especially their electrical resistivity.

Apparently, having knowledge about the electrical characteristics of the Salen-based polymers, would open the windows to the great applications of these electrically conductive compounds in different parts of sciences such as electrochemical capacitor, batteries, etc.^9,10^ About these devices, the electrical resistivity of these conductive polymers has significant influence on different electrical parameters such as the energy density, power density, etc.^10^ This is because, the resistivity majorly affects the energy level density of batteries through controlling the charge and mass transfer processes during reversible redox half reaction(s)^10^. However, this phenomenon is beyond its substantial stimulus on the dielectric constant of electrochemical capacitors to reach an electronic device with maximum power density through the fabrication of supercapacitor^11^.

At the ideal conditions, the intermediate combination of these two factors is connected in a single device called electrical double-layer capacitors (EDLCs)^10,11^, whose properties are attributed to obedient control in the electrical sequences of the conjugated polymers. However, there are significant limitations in the synthesis of these ideally capacitive material with acceptable battery-energy level and acceptable cycle life^5^.

It seems that, focusing on conductive and nonconductive molecular micro/ nano wires can solve the current existing problems. Conductive molecular nano/micro wires, due to possessing high electrical charge, are considered as good candidate for fabricate different power sources. About these system, the electrical net charge is mostly controlled and managed by different strategies such as formation of Zwitterion, ion-paring, presence of electronegative elements in the molecular structure, coordination with metal cations and resonance^3^. Among these strategies, the limit of the electrical charge can be controlled via adjusting the strength of the hybrid renounce. This is often occurred via chemically or electro-chemically conjugate the π orbitals in the double bonds of the selected molecule. At this condition, accessing to a reliable protocol for precise controlling the limits of the conjugation of the π orbitals, can open a light view point for the scientists to control the dielectric constant of some different compounds such as Salen during formation of for instance a capacitor with high electrical capacity.

To reach this aim, selection of Salen is attributed to its special characteristic such as i) its simple synthetic process, ii) process of wide resonance form in its structure, and ii) its lability to coordinate with different cations. However, these extent and application of this protocol can be promoted when accessing to an array of the self-assembled molecular wires with special characteristics^2–4^. Obviosity, layer-by-layered self-assembly of the molecular wires can direct the researchers to reach these ideal conditions. For this purpose, hereby, in this study, for the first time, a novel electro-synthetic methodology is introduced to synthesize conjugated/unconjugated Salen-based polymer nanostructure, along with evaluation of its application as pseudo—supercapacitor during the optimization process.

## Experimental

Detailed introduction about all the adopted reagents and solutions were reported in the “*Supplementary Manuscript*” (See *Supporting Information*, Sect. 1.SP.). In addition, Sect. 2. SP (See Supporting Information) show the apparatus including the selected three-electrode system, electrochemical modes such as cyclic voltammetry (*CV*) and electrochemical impedance spectrometry (*EIS*) as well as detail characteristic about the spectroscopic systems.

In this experiment, Salen and Salophene were utilized as the selected probes, whose synthetic procedure were reported in detail in the “*Supplementary Manuscript”* (See Sect. 3.SP.)

All reagents, solutions, and chemical and electrochemical synthesis of Salen and Salophene are given in supporting information (*SI*) file (See *Supporting Information*, Sect. 1.SP.).

## Results and discussion

### Electrosynthesis of Salen polymer: effect of successive cyclic voltammetry

**Figure 1:**
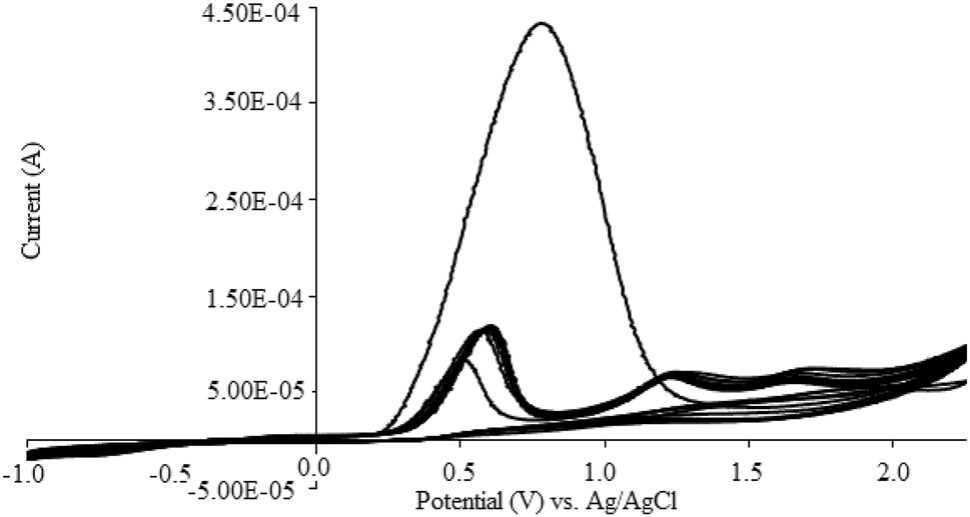
Continuous cyclic voltammograms (CVs) of Salen monomer (0.04 M) in acetone at potential ranging between − 1.0 to + 2.25 V and a scan rate of 100 mV s^−1^ at pH > 13.

The continuous (Successive) CV of the Salen monomer (0.040 ± 0.001 mol L^−1^) during the electrosynthesis of the conjugated polymer (See Supplementary Manuscript, Sect. 4.SP.) at potential window ranged between − 1.00 and + 2.25 V (± 0.01, vs. Ag/AgCl, sat’d Cl^-^) and a scan rate of 100 mV s^−1^ has been shown in Fig. 1. As shown, observation of a sensitive peak current at the first scanning cycle at + 0.70 ± 0.01 V (vs. Ag/AgCl) was related to the anodic electrosynthesis of the Salen-based polymer. However, during the continuous CV from the second cycle, a significant decrease was observed in the anodic peak current (Fig. 1). As clearly evaluated, the more was the number of cycles, the lower was the anodic peak intensity after the first CV cycle. This phenomenon was attributed to the electrosynthesis of conductive Salen-based polymer (Fig. 1), which aggraded with the results reported in the previously published articles^3^. Although, significant reduction in the electrical peak current from the 2nd CV cycle pointed to low electrical conductivity of the glassy carbon (*GC*) electrode during modification with the Salen polymer (Fig. 1SP). However, observation of the third peak at 1.75 ± 0.01 V (vs. ag/AgCl) was ascribed to the oxidation acetone as the co-solvent on the electrode surface that was probably electro-catalyzed by the modified Salen-based polymer.

**Figure 2:**
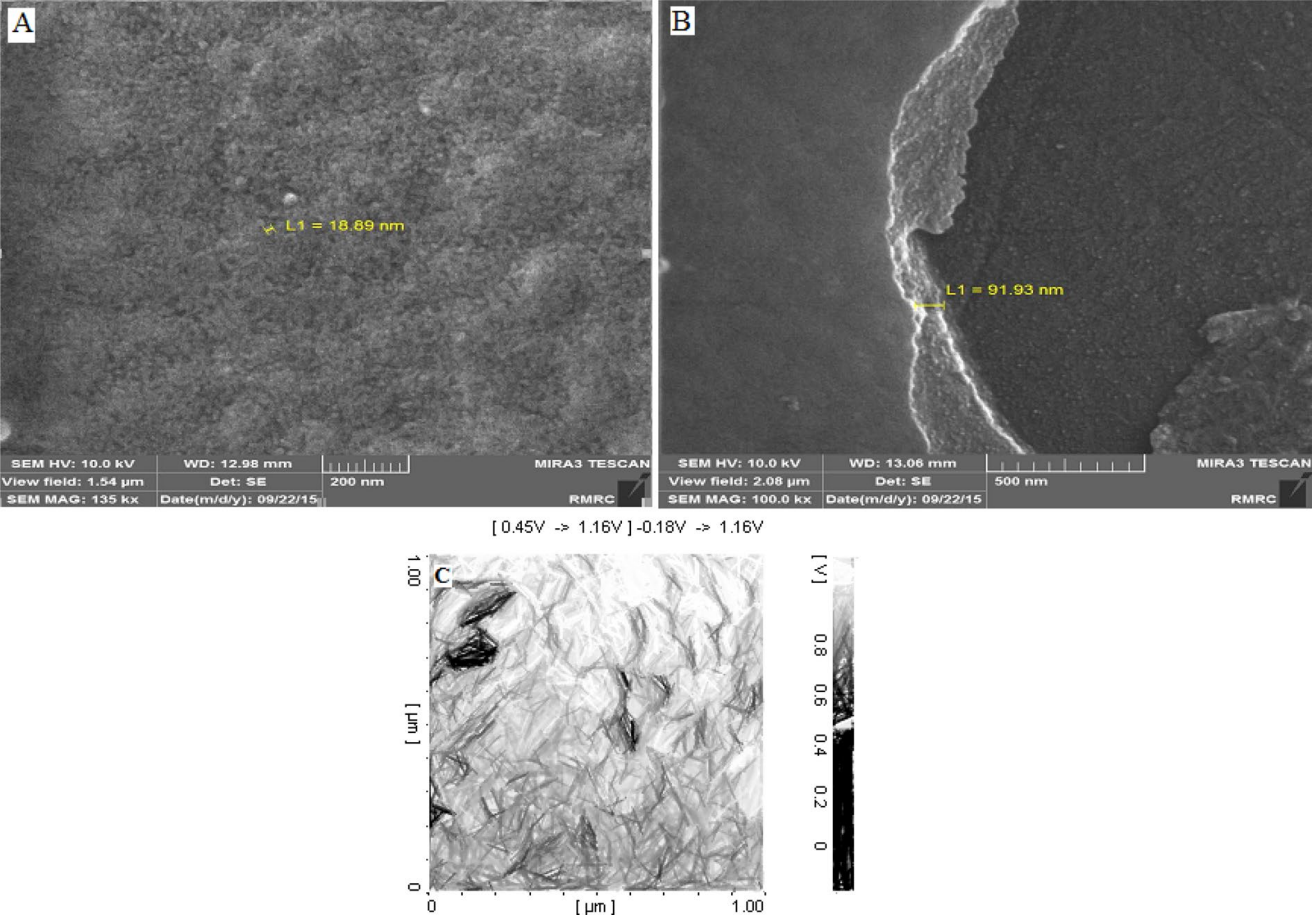
Images of (**A**, **B**) FE-SEM and (**C**) HR-AFM of electro-synthesized Salen-based polymer as nanowires on the surface of GC electrode during using K^+^ (0.01 mol L^−1^).

The morphology of the synthesized conjugated Salen-based polymer was also evaluated using field emission-scanning electron microscopy (*FE-SEM*) and high resolution-atomic forced microscopy (*HR-AFM*) as shown in Fig. 2. As shown, the thickness of the synthesized polymer layer was estimated to ~ 90 nm according to the FE-SEM images (Fig. 2A and B). Also, high active surface area of the synthesized polymer was confirmed based on the defect (roughness) observed using the HR-AFM image (Fig. 2C). This observation pointed to the thin film of the electrosynthesized Salen-based polymer.

Though, at the initial point of view, this phenomenon seemed to be a serious challenge, regarded with significant limitations in the charge and mass transfer processes in this electrochemical systems. Conversely, this could be simply solved via coordinating the electro-synthesized Salen-based polymer with a labile metal ion such as Cu^2+^, Fe^3+^, etc.^10,11^ These results has been related to the probable difference(s) in the resistivity of the synthesized polymer along different sequential CV cycles.

**Figure 3:**
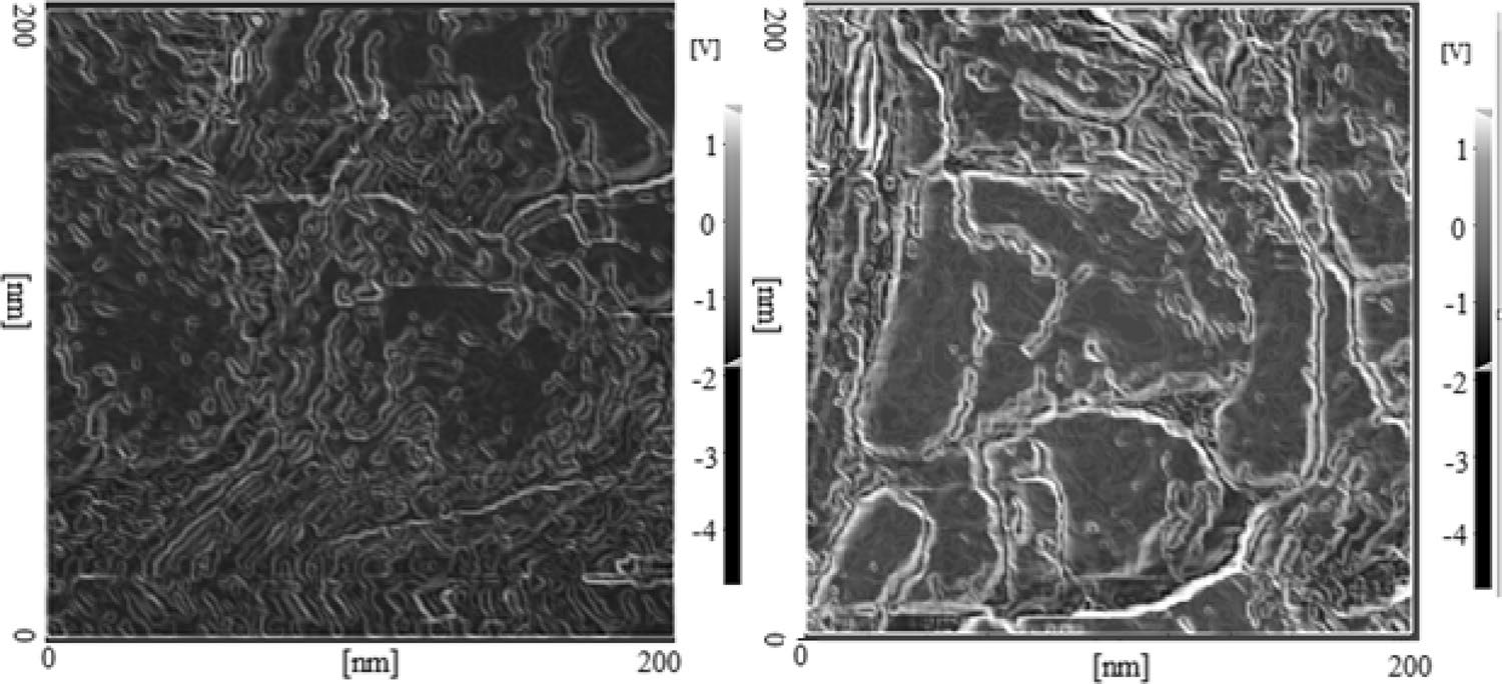
HR-AFM images of conjugated/unconjugated Salen-based polymer as conductive nanowires.

In this study, surprisingly, elimination of the labile transition metal ion(s) from the electrolyte caused to observe a new anodic peaks from the 2nd CV cycle at potential of + 1.20 ± 0.001 V (vs. Ag/AgCl). This phenomenon was attributed to further oxidations of the electro-synthesized Salen-based polymer and formation of conjugated Salen-based polymer as the synthetic metals. Consequently, repeating the CV cycles provided alternative (layer-by-layered) synthesis of the conjugated/unconjugated Salen-based polymer. At this condition, this process led to have well-defined polymeric nanowire, which played role as a sensitive pseudo-supercapacitor. The morphology of the electrosynthesized Salen-based polymer as a nanowire with the average diameter of ~ 10 nm is clearly demonstrated according to the HR-AFM images, shown in Fig. 3.

### Characterization of conjugated/nuconjugated Salen-based polymer

To have further confidence about this phenomenon, it was decided to further characterize the formation of the conjugated/unconjugated polymer that are reported in detail in the following sections:

#### Salophene as another selected probe

To further evaluate the C=C band formation during the oxidation process, Salophene was selected as an appropriate model (See Supporting Information, Sect. 5.SP.). This selection was based on the presence of phenolic functional groups and conjugated structure in its molecules, due to its structural similarity to the conjugated Salen-based polymer. Therefore, this compound was considered as an appropriate model (Selected probe) to evaluate the possibility of the formation of C=C during the synthesis of conjugated polymer through the electro-polymerization process. Detail study about the formation of C=C bond by the electrosynthesis process has been evaluated in detail based on the FT-IR analysis in the Supplementary Manuscript (See *Supporting Information*, Sec.: 6.SP.).

According to the results (Fig. 1), during focusing on the for the 1st and 2nd CV cycles under similar conditions, a significant change was observed in the number, and potential of the anodic peaks of the Salophene, compared to those exbserved for the Salen-based polymer. Based on the results (Fig. 1), disappearance of the 2nd anodic peak clearly exhibited the correlation of this peak to the formation of C=C bond in the Salen polymerization process. This observation was therefore considered as an acceptable model for approving the possible formation of the C=C bond during the synthesis of the Salen-based polymer by the recommend procedure.

### Proposed behavior

**Scheme 1:**
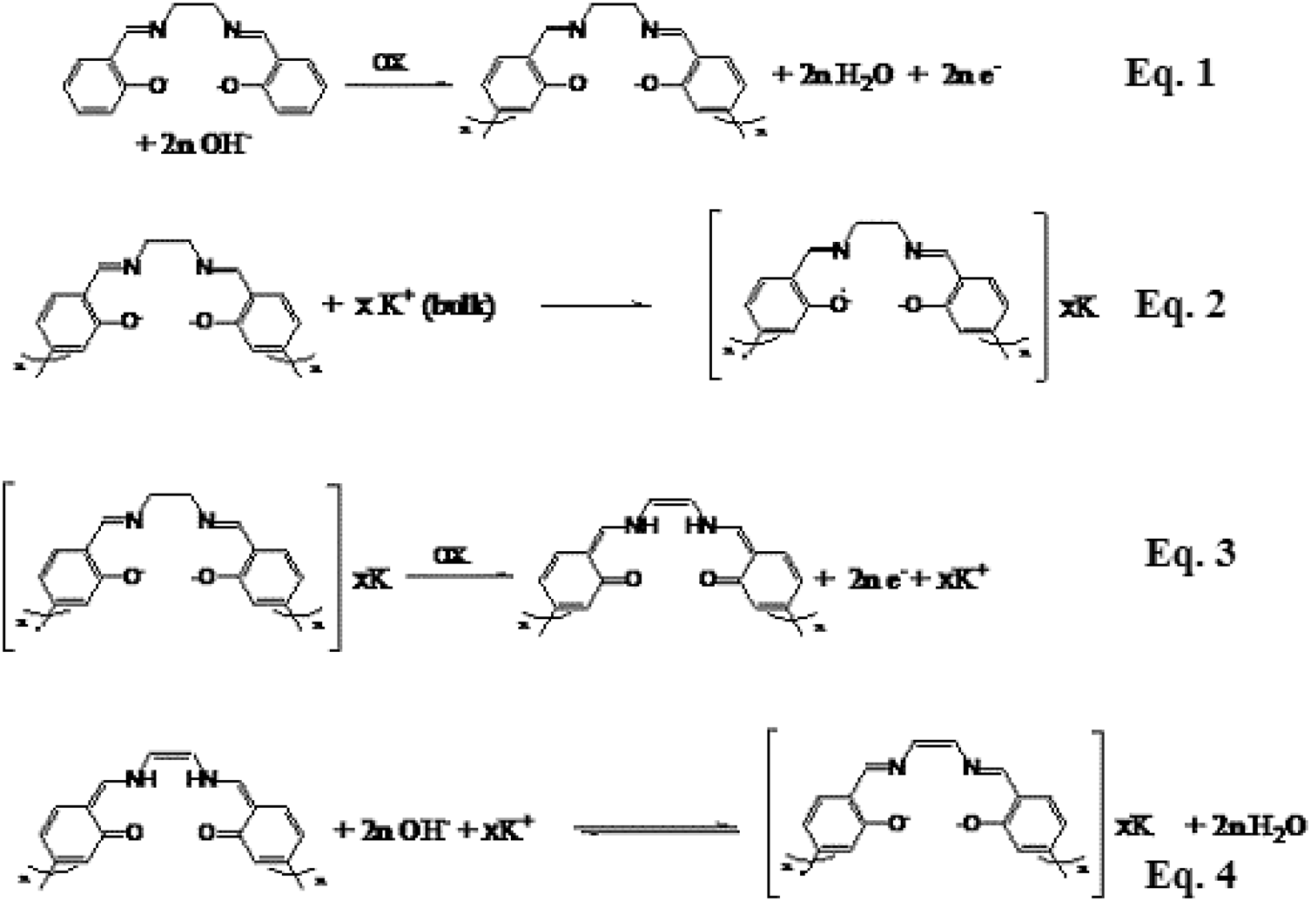
Proposed behavior (strategy) of the electro-polymerization of the conjugated/unconjugated Salen-based polymer as nanowires.

During the electro-polymerization of the Salen-based polymer (Scheme 1), different changes were occurred on the structure of the synthesied polymer. As explained before, regarding the CVs (Fig. 1), three independent anodic peaks were observed at potentials of + 0.50, + 1.25 and + 1.75 (± 0.01) V (vs. Ag/AgCl), See *Supporting Information*, Sec. 7.SP. The first two oxidation peaks were therefore attributed to the formation of the Salen-based polymer (Scheme 1), followed by its further oxidation during formation of C = C and oxidation of the phenol group, respectively. On the basis of the CV, the last peak was attributed to the oxidation of the co-solvent (acetone) at strong basic condition that caused to the formation of acetic acid and carbon dioxide. All parts of these reactions were shown in Scheme 1.

### Application

**Figure 4:**
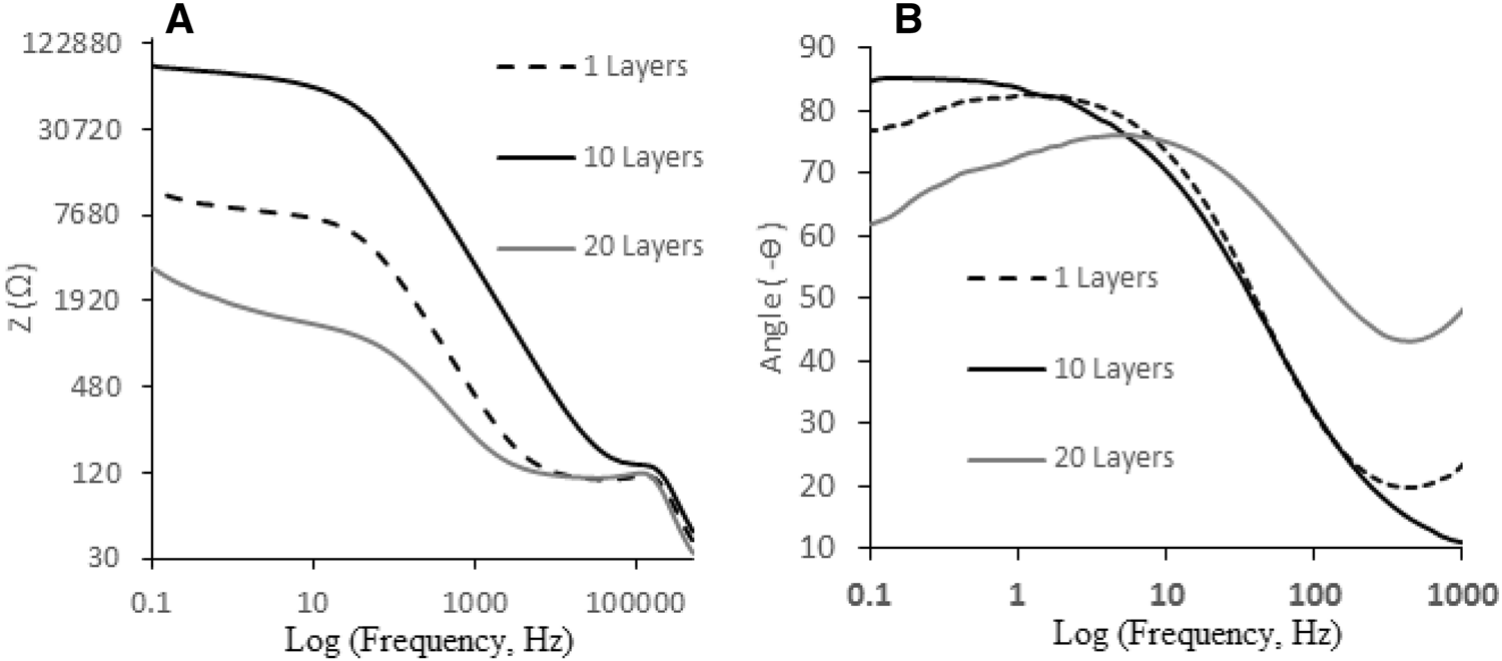
Diagrams of real impedance and phase angle vs. frequency (Bode and angle-frequency plots of conjugated/unconjugated Salen-based polymer as nanowires modified on the surface of GC electrode through the electrosynthetic process.

**Figure 5:**
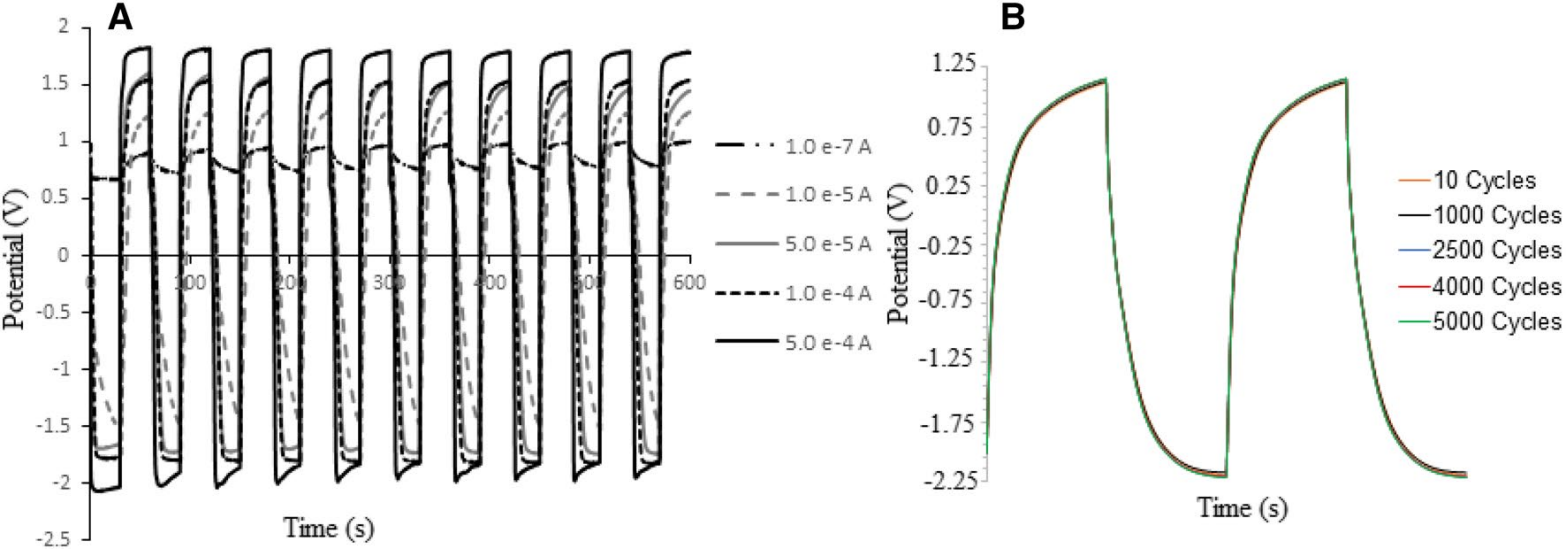
Diagrams of potential vs. time (charge/discharge-time curves) of Salen polymer as nanowires modified on GC electrode at different applied electrical currents (ranged between 1.0 × 10^−7^ to 5.0 × 10^−4^ A) and various CV cycles up to (**A**) 500 and (**B**) 5000 cycles.

In the following sections, potential of the electro-synthesized layer-by-layered (self-assembly) conjugated/unconjugated Salen-based polymer as electrochemical capacitor has been evaluated in detail (See *Supporting Information*, Sec. 8.SP.). As shown (See Supporting Information, Sects. 9.SP–13.SP.), each CV (Figs. 2SP and 3.SP.), photographic images (Fig. 4.SP), FT-IR characterizations (Fig. 5SP), XPS analysis (Fig. 6SP) strongly revealed the formation of conjugated/unconjugated Salen -based polymer.

Optimized effects of the two-mixture solvent, pH, ionic strength, and potential window during the electrosynthesis of the Salen polymer, Salen concentration and coordination compounds have been presented in detail the SI file (See Supporting Information, Sect. 14.SP–17.SP). Detail discussion about the optimization and characterization has been shown in detail in Figs. 7.SP–12.SP.

#### Electrochemical impedance spectrometry (EIS)

The electrical resistance of the polymer was measured using an ohmmeter (Fluke) that was estimated to be 1.3 ± 0.2 (n = 5) kΩ cm^−1^. In addition, the electrochemical impedance spectroscopy (EIS) of the synthesized conjugated/unconjugated polymer at different scanning cycles have been evaluated in detail based on the Nyquist plots shown in Fig. 11SP (See Supporting Information, Sect. 18.SP). The calculated electrochemical parameters such as R_s_, C_dl_, R_dl_, and so on have also been reported in Table 1.SP.

On the basis of the results, significant changes were observed during the continuous CVs, which pointed to the formation of the conjugated/unconjugated polymer as conductive/nonconductive moleclular wire. The electrical capacity behavior of the synthesized conjugated/unconjugated polymer has clearly been evaluated based on the Bode plot (diagram between real impedance, -Z, vs. frequency) as shown in Fig. 4A. As shown, the electrical capacity of the synthesized polymer was observed only at the 10th CV cycle. At CV cycles higher and lower than 10th, the behavior of the polymer played therefore role as a simple resistor.

The electrical parameters related to the 10th layer of the conjugated/unconjugated polymer have also been reported in Table 2.SP. The reported data was estimated based on the equations reported in Ref.^12^ As shown, the electrical capacity of this polymer was not comparable with the GC electrode and also with that synthesized during repeating the CV mode lower and higher than 10th cycle. The supercapacity behavior of this conjugated/unconjugated polymer was therefore obvious. Therefore, the EIS was considered as an acceptable probe to evaluate the super-capacity behavior of the electrosynthesized conjugated/unconjugated Salen-based polymer.

#### Frequency-dependent supercapacitor

Regarding the EIS, the frequency of active mode for the introduced pseudo-supercapacitor was estimated between 0.0 and 500.0 kHz was reported in Fig. 4B. As exhibited, negative and sharp slope of the diagram clearly pointed to the supercapacity behavior of the polymer. According to Fig. 4B, phase angle was very close to 90°, which absolutely pointed to the ideal pseudo-supercapacitive behavior of the conjugated/unconjugated polymer.

#### Chrono-potentiometry

Another effective technique for characterization of the supercapacitive behavior of the conjugated/unconjugated polymer is the “*Chrono-potentiometry*”. The applicability of this technique was related to the storage of electrical charges on the surface of the synthesized conjugated/unconjugated Salen-based polymer as molecular wire. According to the results (Fig. 12SP, See *Supporting Information*), the sequence of the changes in the electrical current was enhancing. This was related to the effective role of the electrical potential on the charging of the supercapacitor.

However, observation of some fluctuations (perturbations) in the diagram probably pointed to the change in the electrical conductivity of the electrode during the sequential and alternative formations of conjugated/unconjugated Salen-based polymer. In another word, as soon as the electrical conductivity of the Salen polymer on the GC electrode surfer was promoted via formation of conjugated Salen-based polymer, the conditions for the further electrosynthesis and the immobilization of larger amounts of Salen-based polymer on the GC electrode surface was enhanced.

In addition, owing to the significant difference between the anodic peak currents at the 1st and the 2nd CV cycles, sequential fractions of the Salen-based polymer were alternatively changed to the conjugated/unconjugated polymers as both nanowire and dielectric during the formation of C=C bond. This phenomenon indirectly revealed the suggested morphology proposed for the electrosynthesized conjugated nanowire (as the conductive nano plates) and unconjugated ones (as the dielectric) of the fabricated Salen-based supercapacitor. These results again revealed the electrical capacity behavior of the synthesized self-assembly conjugated/unconjugated polymer by the recommended procedure.

### Figures of merit: electrical charge/discharge

Regarding to the information reported in Ref,^10,11^ the electrical charge/discharge behavior of the synthesized conjugated/unconjugated nanowire was evaluated using the “*Chrono-potentiometry*” at different electrical currents up to 5000th cycle currents ranged from 1.0 × 10^–7^ to 5.0 × 10^–4^ A. Based on the chrono-potentiograms (Fig. 5A and B), reproducible charge/discharge conditions were exhibited at potential values from -2.00 to + 1.75 V (± 0.01, vs. Ag/AgCl). For this purpose, maximum possible rate was selected for this supercapacitor depending on the time constant of the synthesized pseudo-supercapacitor as large as below 1.5 ms (Fig. 5). In addition according to the EIS tests, the polymer films were accepted as an acceptable supercapacitor with the real capacity of 3110 ± 6 (n = 3) C g^−1^.

The pseudo-capacitor behavior of the electro-synthesized self-assembled nanowire was attributed to different phenomena, especially (1) roughness of the polymer (Fig. 2), (2) presence of multiple functional groups as edge planes and active sites in the polymer matrix (Fig. 3SP), (3) charge transfer along the polymer chain due to the conjugation form of the polymer (Fig. 1), (4) sequential electrosynthesis of conductive polymer as electrical plates and non-conductive polymers (as dielectric) with high active surface area, resulted in the synthesis of arrays of several supercapacitors (Fig. 12SP), (5) coordination of the conjugated Salen-based polymer and monomer with K^+^ (Fig. 4SP) and finally (6) layer-by-layered formation of conjugated/unconjugated polymer (Fig. 1SP).

However, it should be noted that, to estimate the amount of specific capacitance, it was impossible to evaluate the real quantity of the active surface area using standard probes such as Fe(CN)_6_^3−/4−^,^10,11^ because of the presence of nano-film during the formation of the pseudo-supercapacitor. Impossibility to access the precise value of the diffusion coefficients of the standard probe inside a two-mixture solvent such as mixture of H_2_O and acetone is also another reason for unfeasibility to estimate the real active surface area. In addition, the very thin layer as well as small amounts of the electrosynthesized conjugated/unconjugated Salen-based nanowire, measuring of the active surface area using some physio-chemical analyses such as BET was also cancelled. That was why, in this study, it was impossible to quantitatively estimate the specific capacitance for the introduced supercapacitor. All these results point to the acceptable behavior of the synthesized Salen-based polymer as a novel supercapacitor.

## Conclusions

In this experiment, a novel electrosynthetic method was introduced to synthesize the conductive/nonconductive Salen-based polymeric nanowire using Salen as a precursor. These properties were also attributed to the lack of the utilization of any reversible electroactive metal ions such as Ni2^+^and Fe^2+^ during the formation of complex with the polymer. Compared to the previously reported articles that mostly adopted metal/Salen complex as the precursor of the Salen-based polymer inside non-polar solvents such as DMF and DMSO, the use of free Salen as a monomer led to a thin-film Salen polymer with conjugated/unconjugated matrix. This study was therefore considered as a window for solving the main limitation of the previously reported articles on the electrosynthesis of the Salen-based polymer^12–18^. To the best of knowledge, about these reports, the electroactive behavior of the coordinated metal ions perverted the generation of the conjugated/unconjugated polymer. Whereas in this study, the charge transfer behavior from one side led to have a conjugated polymer and from the other hand, the synthesized unconjugated thin films of the Salen-based polymer behaved as a real dielectric material. This process simply majored to have several (10 sequnetaila layer-by-layer) electrical supercapacitor. To the best knowledge, this study is considered as the first report in which the electrochemical supercapacitor behavior of conjugated/unconjugated Salen-based polymer is evaluated with acceptable figures of merit.

## Supplementary Information

Supplementary Information.
