# Supplementary material for: Alternate layer by layered self assembly of conjugated and unconjugated Salen based nanowires as capacitive pseudo supercapacitor
Source: Sci Rep. 2021 Sep 22;11:18768. doi: 10.1038/s41598-021-98288-y (PMC8458273; doi:10.1038/s41598-021-98288-y)
Supplement: Supplementary file 1 — Supplementary Information. [file 41598_2021_98288_MOESM1_ESM.docx]

**Supporting Information**

**Related to**

**Alternate layer by layered self assembly of conjugated and unconjugated Salen based nanowires as capacitive pseudo supercapacitor**

**Mohammad Mahdi Doroodmand*and Sina Owji**

Department of Chemistry, College of Sciences, Shiraz University, Shiraz 71454, Iran.

***Corresponding Author**: Doroodmand@shirazu.ac.ir, [Doroodmand@yahoo.com](mailto:Doroodmand@yahoo.com), Tel: +098-713-6137152, Fax: +098-713-6460788.

**1.SP. Reagents and solutions**

All the chemical reagents were from their analytical grades. Inorganic compounds such as KCl, NaCl, NaOH and LiCl with the purity percentage of >99.0 %, as well as commensal HCl (32.0 %, w/w), and potassium salts of Fe(CN)_6_^3-/4-^ (> 99.5 %) were all from the Merck Company. Analytical grades of non-aqueous solvents like methanol (CH_3_OH, 99.9 %), ethanol (C_2_H_5_OH, 99.5 %,), propanol (C_3_H_8_OH, >99.5 %), N,N-Dimethylformamide (DMF, 99.8 %), Dimethyl sulfoxide (DMSO, 99.9 %), and acetone (C_3_H_6_O, 99.5 %) were also purchased from the Merck Company. Deionized water (Specific conductivity: 1.00 ± 0.01 µS m) was also adopted as solvent that was related to the Fluka Company. To synthesize the Salen monomer, analytical grades of salicylaldehyde (C_7_H_6_O_2_, >99 %) and ethylenediamine (C_2_H_8_N_2_, > 99.0 %,) were related to the Sigma-Aldrich Company. Synthesized Salophene was also adopted as selected probe to evaluate the reliability of the proposed mechanism.

**2.SP. Apparatus**

A three-electrode system consisted of glassy carbon (GC, Metrohm AG Company), Ag/AgCl (Sat’d Cl^-^, Metrohm Company) and a Pt rod (99.996%, i.d.: 2.0 mm, height: 3.0 mm, Azar Electrode, Orumieh, Iran) as working, reference and the counter electrodes, respectively. Electrochemical modes including cyclic voltammetry (CV), electrochemical impedance spectroscopy (EIS) and chrono-amperometry were performed using a Potentiostat–Galvanostat, Metrohm µAutolab ® type Ш. All the experiments were conducted at 25 ± 2 °C. The Fourier transform-infrared (FT-IR) spectra were obtained using Shimadzu (FT-IR, Shimadzu-8300, Markham, Ontario, Canada) spectrometer. X-ray photoelectron spectrometry (XPS, Bestec Instruments, Shimadzu, Canada) was utilized to characterize the synthesized conjugated/unconjugated Salen-based polymer. A ultra-violet (UV-Vis.) spectrophotometer (Pharmacia Biotech, Ultrospec 4000, Canada) was also selected using a 1.0-cm quartz cell to estimate the K^+^/Salen mole ratio in the coordinated compound (complex). Furthermore, a fluorescence optical microscopy (CETI-Magnum T, Shimadzu Company, Canada) was employed to image the synthesized thin nano-film polymer. The apparent structure of the synthesized Salen-based polymer has been evaluated using a scanning electron microscopy (FE-SEM: Mira3-xmu, KYKY-EM3200, 25.0 KV, US) and a high resolution- atomic forced microscopy (HR-AFM, DME-SPM, ScanTool™, 2.0.0.9, US).

**3.SP. Synthesis of Salen and Salophene**

To synthesize the Salen monomer, 20.0 mL of the mixture of acetone:water (80:20, V/V) was selected as solvent. 200.0 mL dried ethanol was added to 0.10 ± 0.01 mole (10.60 ± 0.02 mL) of salicylaldehyde and stirred at room temperature according to a reported procedure.^1^ After that, 0.050 ± 0.001 mole (3.30 ± 0.01 mL) of ethylenediamine was added dropwise during a 5.0-min period. The basicity of the solution was controlled to high strong basic condition using KOH solution (0.01 mol L^-1^, 5.0 mL). The concentration of KCl was also set to 0.040 ± 0.001 mol L^-1^ concentration. After a few seconds (at least 5 s), a yellow precipitation was observed. To fulfill the reaction, the reagent was stirred at room temperature for 30.0 min. Subsequently, inside a lab digital thermostatic water bath (Tg-2050 Series Rt-200, GH-15A, China), the suspension temperature was set to the 0.0 ± 0.3 ^o^C. The precipitation was then separated by filtering through a paper filter (Average mesh size: 300 nm, Porosity: 6.0 µm, Fisher Scientific, USA). Afterward, the precipitation was washed using cooled ethanol (10.0 mL, <4 ^o^C) for three times. Finally, the solid product was dried at 50.0 ± 0.3 ^o^C using a thermal/drying oven (DHG-9023A, China) at air atmosphere during 30.0 min time interval.

To synthesize the Salophene (0.040 ± 0.001 mol L^-1^) monomer, similar to the previous section, 200.0 mL dried ethanol was added to 0.10 ± 0.01 mole (10.60 ±0.01 mL) of salicylaldehyde and stirred at room temperature using a selected procedure ^12^. Then, 0.050 ± 0.001 mole (5.400 ± 0.001 g) O-phenylenediamine was slowly added during a 10.0-min interval. The synthetic process of the Salophene was then completed according to the procedure recommended for that of the Salen polymer.

**4.SP. Self-assembly of salen-based conjugated/unconjugated nanowires**

Continuous CV modes during at least 20 sequential cycles simply caused to the alternative (layer-by-layered) conjugated/unconjugated Salen-based polymer as conductive/nonconductive nanowires. Finally, the surface of the GC electrode was washed using a lab-prepared triple-distilled water medium (5.0 mL) and dried by exposure to the air along 2.0 min using a previously published method.^2^ The GC electrode was finally selected for examining the pseudo-supercapacitor behavior.

**5.SP. Electrosynthesis of salophene-based polymers**

To synthesize the Salophene-based polymer, Salophene monomer was adopted as the selected probe. The same electrode system was again considered as the polymer synthesis. The CV mode was also utilized during applying potential values between -1.00 and +2.25 V (±0.01, vs. Ag/AgCl, sat’d Cl^-^) at 100 mV s^-1^ scan rate using alkaline acetone/water (80:20, V/V, 10.0 mL) as electrolyte with at least 20 continuous (repetitive) CV cycles. This procedure was also similar, partially, to that reported for the synthesis of the Salen-based polymer.

**6.SP. FT-IR/gravimetric analyses**

Along finalizing the electro-synthesis process of the conjugated/unconjugated Salen-based polymer on the surface of GC electrode, the thin film conjugated Salen polymer was tightly immobilized on the surface of the GC electrode. About the FT-IR and the gravimetric analyses, it was impossible to separate the polymeric film for characterization using the FT-IR spectrometry. For this purpose, an electrochemical technique was adopted via formation of O_2_ at the anodic wall of the electrode system during applying a constant potential value equal to +2.25 ± 0.01 V (vs. Ag/AgCl) for 2.0 s in a 0.01 mol L^-1^ KOH solution as electrolyte. A yellow-brown thin film polymer was then removed from the electrode surface and floated on the solution’s surface.

After drying the sample at ~60 ^o^C for 30 min inside an oven, the sample was mixed with about 100-fold excess of dryad KBr powder (>99.96%, Merck Company) and analyzed using the FT-IR spectrometer. The same procedure was adopted for weighting the electrosynthesized conjugated/unconjugated Salen based film using a Metler gravimeter for estimation of the real capacitance value with C g^-1^ unit using the chrono-potentiometry method (Fig. 5).

**7.SP. Statistical analysis**

The optimization process was based on the one-at-a time method. Each reported data was based on the average of at least three replicate analyses. In addition, the uncertainty of each datum was reported based on the ±standard deviation (±SD).

**8.SP. Electrochemical capacity measurement**

To estimate the electrochemical capacity of the synthesized conjugated/unconjugated Salen based polymer, the electrical charge/discharge parameters were evaluated using chronopotentiometry at different electrical currents, ranged between 1.0×10^-7^ and 5.0×10^-4^ A. On the basis of the chrono-potentiogram, reproducible charge/discharge process was occurred at potentials between -2.00 and +1.75 V (±0.01, vs. Ag/AgCl). The electrochemical capacitive behavior of the thin-film polymer was also evidenced by EIS using the Nyquist plots.

**9.SP. Effect of potential window during the electrosynthesis of salen polymer**

Another important point related to the electrosynthesis of the Salen polymer was the necessity to sweep the applied electrical potential to the negative potentials before the observation of any anodic peak current(s). To evaluate the amount of the applied negative potentials, the synthesis of Salen solution (0.040 ± 0.001 mol L^-1^) was studied at a scan rate of 100 mV s^-1^ at different potential windows as shown in Fig. 1SP. As can be seen in Fig. 1SP, the more negative was the potentials, the more sensitive anodic currents were evaluated.


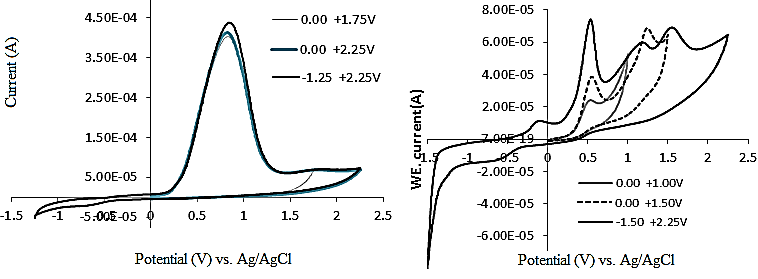


**Fig. 1 SP. |** Continuous cyclic voltammograms (CVs) showing the effects of different electrical potential windows applied to the electrode system during Salen (0.040 ±0.001 mol L^-1^)-based electro-polymerization process using acetone:water as two-mixture solvent at A) 1^st^ and B) 20^th^ scan cycles at a scan rate of 100 mV s^-1^ at strong basic condition using KOH solution (0.01 mol L^-1^, 5.0 mL).

In this experiment, to reach the highest anodic peak current during the electrosynthesis process, maximum negative potential (-1.00 ± 0.01 V, vs. Ag/AgCl), which was close to the cathodic reduction wall of H_2_O (i.e. -1.0 V, vs. Ag/AgCl) was selected by the CV mode. Moreover, a potential window between -1.00 and +2.25 V (±0.01, vs. Ag/AgCl) was selected. This potential window was considered as the best potential range for distinctly controlling the sequence of different oxidation peaks at the 1^st^ and 2^nd^ CV cycles during the synthesis of conjugated/unconjugated Salen-based polymer as the synthetic nanowires. This potential range, not only controlled the layer-by-layered formation of conjugated and unconjugated synthetic nanowires, but also cancelled some limitations such as kinetic polarization of the working electrode during controlling the mass/charge transfer processes of the Salen monomer in the electro-synthesis process.

**10.SP. Effects of scan rate**

Electrochemically, the difference between a battery and a supercapacitor is related to the difference between the mechanisms of the mass/charge transfer process. About both categories based on the kinetic information, the electrical current vs. scan rate (v) is obeyed for the equation stated as i=a.v^b^ .^3,4^ Whereas about the batteries, the mechanism of the charge transfer process on the surface of an adequate battery material as the working electrode is based on the diffusion process, at which the b value is close to ~0.5 .^4^

In another word, linear correlation is existed between the electrical current vs. square root of scan rate (v^1/2^). Whereas in the EDLCs, the stored charge is based on the adsorption of the electrolyte ions on the surface of the electrode material. At this condition, the electrical current is linearly correlated to the scan rate (v). This process therefore causes b value to be approached towards 1.0.^3, 4^ In this system, the effect of scan rate was evaluated in detail. For this purpose, the correlations between the peak currents vs. each v and v^1/2^ showed correlation coefficient (R^2^) the same as 0.9542 and 0.9675, respectively. As shown, presence of partial linear correlations revealed to the mass transfer mechanism, which obeyed from both diffusion and adsorption processes. However, partially more linearity, exhibited between the peak currents vs. the v^1/2^ pointed the importance of the mass transfer based on the diffusion process. These results clearly pointed to both charge transfer as well as adoption on the surface of the working electrode.

For the future evaluations of the scan rate effects on the electrosynthesis of conjugated/unconjugated Salen-based polymers, the electrosynthesis was studied at two different scan rates such as 100.0 and 400.0 mV s^-1^. The voltammograms have been shown in Fig. 2SP. Compared to the voltammogram related to 100 mV s^-1^ scan rate, the anodic peak current related to the anodic polymerization of the Salen at 400 mV s^-1^ scan rate was considerably higher than that observed in the 1^st^ CV cycle.


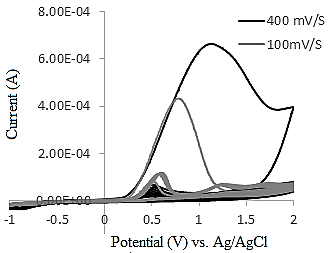


**Fig. 2 SP. |** Continuous cyclic voltammograms (CVs) showing the effect of scan rate on the Salen (0.040 ±0.001 mol L^-1^)-based electro-polymerization process using acetone:water as two-mixture solvent at potential ranging between -1.00 - +2.25 V (±0.01, vs. ag/AgCl) and a scan rates of 100 and 400 mV s^-1^ at strong basic condition using KOH solution (0.01 mol L^-1^, 5.0 mL).

However, about the 400 mV s^-1^ scan rate at the 1^st^ CV cycle, the anodic peak was positioned at +1.20 ± 0.01 V (±0.01, vs. Ag/AgCl) potential. This peak was considered as a combination of the anodic polymerization and the anodic oxidation of the Salen-based polymer during the formation of conjugated/unconjugated polymeric structure. Whereas, this value was estimated to +0.70 ± 0.01 V vs. (Ag/AgCl) potential at 100 mV s^-1^ scan rate with a better peak shape. This effect was attributed to the irreversibility of the anodic polymerization process, which provided more positive over-potential at higher scan rates.

In another word, in spite of the effective role of the scan rates on the anodic peak currents, this factor was significantly higher for the CV, evaluated at the selected scan rate, compared to higher scan rates such as 400 mV s^-1^ scan rate. The effect of peak current was probably correlated to higher electrical conductivity of the synthesized Salen-based polymer during the formation of conjugated structure. Effect of scan rate clearly revealed the effective role of the scan rate of the couple of the chemical and electrochemical processes during the formation of conjugated/unconjugated Salen-based polymer. Consequently, scan rate was considered as an important factor during the formation of conjugated/unconjugated Salen-based polymer.

**11.SP. Selection of conjugated Salen-coordinating cation**

To evaluate the effects of cations through the polymerization of the Salen-based polymer, the coordinating behavior of various cations such as K^+^, Na^+^, Li^+^, Ca^2+^, Ba^2+^, Mg^2+^, Al^3+^, etc. were individually evaluated in detail under similar conditions. However, insoluble precipitations were observed during the formation of Salen complexes with Ca^2+^, Ba^2+^, Mg^2+^, Al^3+^, etc, as most of these cations with two or three capacities were insoluble in the acetone: H_2_O solvent. Consequently, it was only emphasized on the cations with one capacity such as K^+^, Na^+,^ and Li^+^. Fig. 3. SP shows the CVs through the electro-polymerization of the polymer using Salen (0.040 ± 0.001 mol L^-1^) at a scan rate of 100 mV s^-1^ at the strong basic condition, controlled using the same concentrations (0.01 mol L^-1^) of each LiOH, NaOH, and KOH under the similar conditions. As the results, the sequence for the sensitivity of the anodic peak currents was as follows: K^+^>Na^+^>Li^+^.


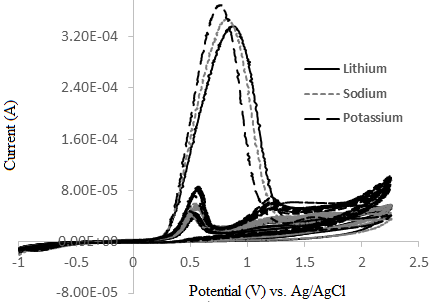


**Fig. 3 SP.** | Continuous cyclic voltammograms (CVs) showing the effect of Li^+^, K^+^ and Na^+^ (0.040 ± 0.001 mol L^-1^) on Salen (0.040 ± 0.001 mol L^-1^)-based electropolymerization process using acetone:water as two-mixture solvent at potential ranging between -1.0 - +2.25 V (±0.01, vs. Ag/AgCl) and a scan rate of 100 mV s^-1^ at high strong basicity using KOH solution (0.01 mol L^-1^, 5.0 mL).

As exhibited, the electrical conductivity of the synthesized Salen-based polymer was directly correlated to the diameter of the cation. This effect was further evaluated via image processing of the Salen polymer-modified GC electrode at such environment with 40.0% humidity that simply generated with water aerosols as shown in Fig. 4SP. Based on the images, the same relationship was detected between the color of the polymer and the atomic spectrum during analysis of K^+^ and Na^+^, whereas no clear color was detected by limiting the potential window between -1.00 and +2.25 V (±0.01, vs. Ag/AgCl).


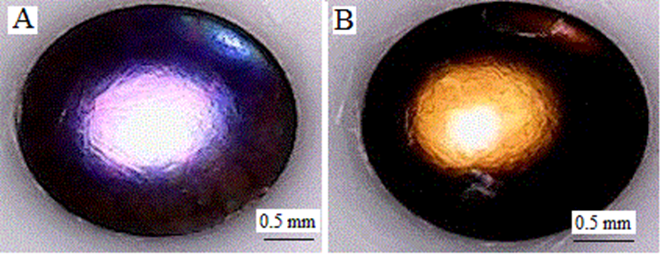


**Fig. 4.SP |** Photographic images of the electrosynthesized Salen (0.040 ± 0.001 mol L^-1^) -based polymeric thin film on the surface of GC electrode during using A) K^+^ and B) Na^+^ (0.040 ± 0.001 mol L^-1^) at 40.0 ± 0.2 % (n=3) relative humidity.

This observation from one side, pointed to the coordination between cations at the negative potentials and from the other hand, revealed the hydrophilicity of the synthesized Salen-based polymer. However, due to the availability of KOH, this reagent was selected to control the acidity and basicity of the electrolyte. This effect was again evidenced via addition of different concentrations of KCl. Further concentrations of K^+^ were optimized through the addition of different concentrations of KCl between 0.00 and 0.05 mol L^-1^. Although the ionic strength of the electrolyte was high enough during partially setting the basicity to pH values higher than ~13, K^+^ had an enhancing effect on the sensitivity of the anodic peak current. This influence was attributed to high conductivity of the Salen polymer/K^+^ resulting in to need less positive over-potential during the electrosynthesis process. It should be noted that, KCl solutions with concentrations above 0.05 mol L^-1^ resulted in having a two-phase solution and precipitation. Consequently, the optimum concentration of KCl was estimated to be 0.040 ± 0.001 mol L^-1^.

Coordination of K^+^ with the Salen- polymer was attributed to the formation of enough negative charge during sweeping the applied electrical potential to the negative potentials. As at the cathodic potential wall of the solvent (H_2_O), the formation of H_2_ bubbles led to the kinetic polarization of the working electrode during the electrosynthesis of the Salen polymer. Consequently, maximum negative potential close to the cathodic potential wall was suitable for the coordination process. As explained in detail in the previous sections, changing the electrical potential to maximum negative value (-1.00 ± 0.01 V, vs. Ag/AgCl) led to have an attraction between K^+^ and the polymer with negative electrical charge. At this condition, the highest anodic peak current was detected during the electrosynthesis process.

**12.SP. Characterization by FT-IR spectrometry**

The FT-IR spectrometry was considered as an excellent technique to estimate the generation of C=C during the electro-polymerization process. To achieve this purpose, the effect of scan rates such as 50.0 and 300 mV s^-1^ was evaluated on the two kinds of Salen-based polymer using the FT-IR spectrometry. As clearly shown (Fig. 5SP), the same behavior was observed during the evaluation of the FT-IR spectrum of the synthesized Salen-based polymers at the two reported scan rates. Nevertheless, observation of an absorption peak at the frequency of ~1385 cm^-1^ pointed to the C=C bond of the benzene cycle. Whereas, formation of aliphatic C=C bond was evidenced according to the absorption peak positioned at ~1442 cm^-1^, which was in good agreement with the vibrational frequency estimated for the C=C based on Ref.^5^


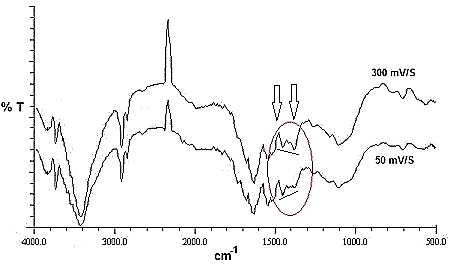


**Fig. 5 SP |** FT-IR spectra of A) Aalen monomer and B) conjugated/unconjugated Salen-based polymer as nanowires. **Conditions**: Acetone :water as two-mixture solvent at potential ranging between -1.0 - +2.25 V (±0.01, vs. Ag/AgCl) and a scan rate of 100 mV s^-1^ at high strong basicity using KOH solution (0.01 mol L^-1^, 5.0 mL).

As shown, reverse relationships between these two absorption peaks pointed to the effective role of low scan rates to the formation of conjugated/unconjugated Salen-based polymer. However, it should be considered that, due to the electrosynthesis and modification of very small quantity of the Salen-based polymer with a thickness of around 90 nm (based on the FE-SEM images, Fig. 2), the intensity of these peaks was small. Although, compared to the absorption intensities at different frequencies, the sequence intensities of the peaks at 1385 and 1442 cm^-1^ were significant. Therefore, at lower scan rate, the formation of C=C bonds was more recognizable. Consequently, FT-IR spectra were again considered as an evidence during the formation of the conjugation process.

**13.SP. XPS spectra**

The XPS was also considered as another effective spectroscopic method for characterization of the synthesized conjugated/unconjugated Salen-based polymer. Fig. 6SP.A shows the XPS spectra of free Salen and the electro-synthesized conjugated/unconjugated Salen-based polymer. Narrow XPS spectra have also been shown in Figs. 6SP.B and 6SP.C. According to the XPS spectrum, the sharp peak positioned at 284.8 eV was attributed to the C_1s_ of the C-C bond.^6^ Whereas, the peak positioned at the 281.2 eV was associated with the C_1s_ of the C=C bond. In addition, the C_1s_ of the C-N bond was positioned at ~288.3 eV. As also clearly shown, according to the XPS spectrum of the synthesized conjugated/unconjugated Salen-based polymer, the peak associated with the C-C bond was completely disappeared. Whereas, the peak related to the C=C bond was significantly enhanced. In addition, a significant decrease was observed in the C-N bond that pointed to the formation of conjugated bonds.


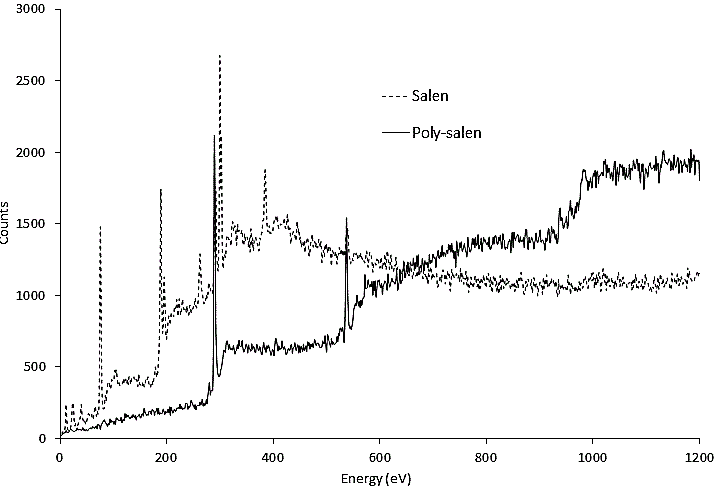


**Fig. 6 SP |** XPS spectra of Salen and conjugated/unconjugated Salen-based polymer as nanowires. **Conditions**: Acetone :water as two-mixture solvent at potential ranging between -1.0 - +2.25 V (±0.01, vs. Ag/AgCl) and a scan rate of 100 mV s^-1^ at high strong basicity using KOH solution (0.01 mol L^-1^, 5.0 mL).

However, due to the dependency of these XPS peaks to the potential windows during sequential electrosynthesis of conjugated and unconjugated Salen-based polymer, therefore self-assembly of the layer-by-layer process was clearly confirmed. All these evidences again pointed to the formation of conjugated/unconjugated Salen-based polymer according to the following proposal behavior.

**14.SP. Optimization**

To have conjugated/unconjugated Salen-based polymer with maximum mechanical and chemical stability, the formation of the self-assembly polymer was also optimized via controlling the related effective factors. Parameters such as the potential window, effect of solvent, solvent ratio, scan rate, effective role of various cations, kind of the working electrode, effect of dissolved oxygen, type of basic solution, the concentration of KOH, and the number of polymeric films were optimized using the one-at-a-time method. Details of the optimization are assessed in the following sections.

**15.SP. Effect of solvent**

As stated in the literature, the electrosynthesis of the Salen-based polymer occurred inside organic solvents such as DMF and DMSO.^2^ The choice of organic solvent was done based on the solubility of metal/Salen complex. For this purpose, effect of different solvents such as propanol, sulfolane, methanol, DMF, acetone, ethanol, diethyl ether, 1-butanol, and isobutanol has been evaluated according to the CVs shown in Figs. 7SP. However, certain solvents such as diethyl ether, 1-butanol and isobutanol did not produce a single phase and transparent solution. As shown (Fig. 7SP), significant enhancements were observed in the anodic peak currents during using solvents such as CH_3_OH, C_2_H_5_OH, and acetone. Consequently, in this study, it was focused on partially polar solvents with high enough polarity such as H_2_O, alcohol, and acetone to wholly dissolve the Salen monomer as the selected precursor for the electrosynthesis process.


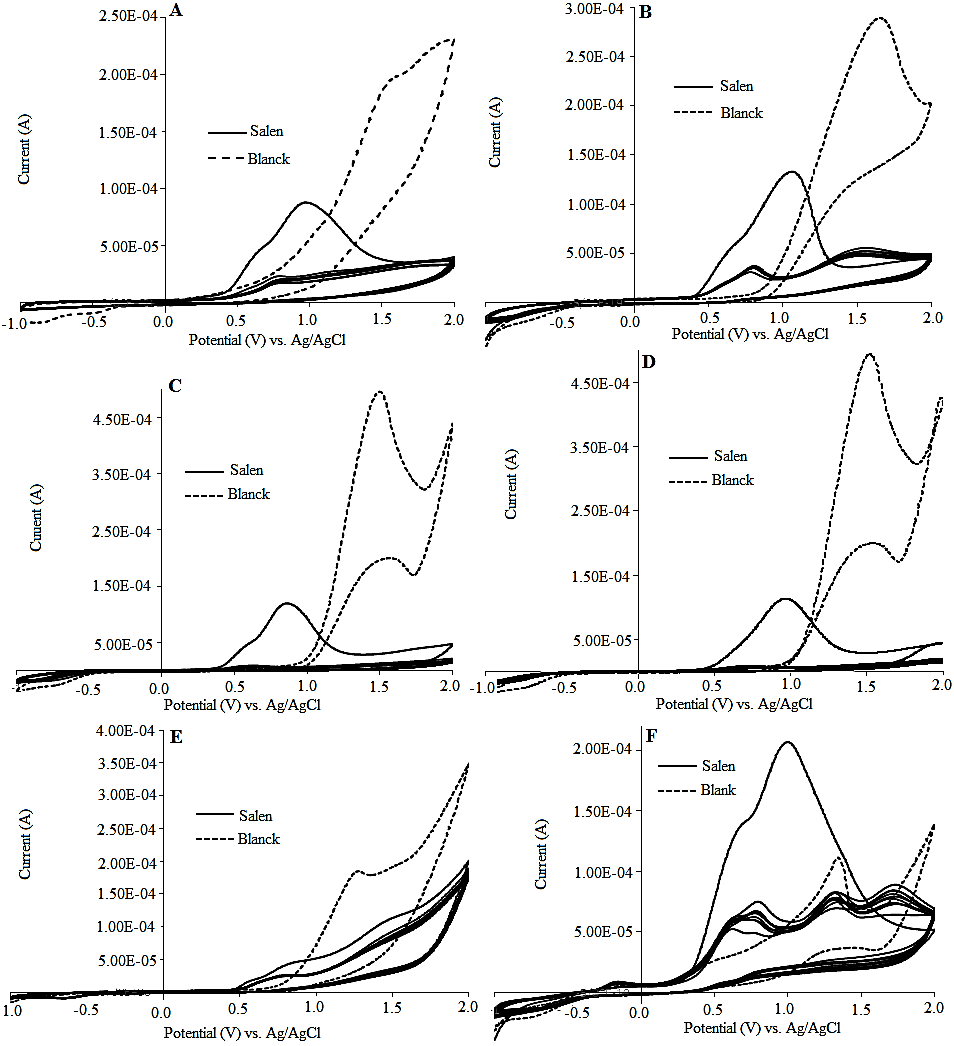


**Fig. 7 SP |.** Continuous cyclic voltammograms (CVs) of Salen-based monomer (0.040 ± 0.001 mol L^-1^) in different solvents including A) Sulfolane, B) Propanol C) Methanol, D) Ethanole, E) DMF, F) Acetone at potential ranging between -1.00 - +2.25 V (±0.01, vs. Ag/AgCl, sat’d Cl^-^) and a scan rate of 100 mV s^-1^ at strong basic condition using KOH solution (0.01 mol L^-1^, 5.0 mL).

Based on the results (Fig. 7SP), the higher was the polarity of these solvents, the lower was the ohmic potential (IR drop) of the electrolyte. This condition therefore resulted in needing less positive electrical potential for the electrosynthesis of the conjugated/unconjugated Salen-based polymer. There was a direct relationship between the anodic current and the amount of electro-synthesized conjugated/unconjugated Salen-based polymer. This phenomenon also exhibited the effective role of these solvents for the promotion of the mass and charge transfer process during the electrosynthesis of the self-assembled polymer. More sensitive signals were also observed during the use of acetone in comparison with alcohols. Therefore, acetone was selected as an appropriate solvent.

**15.1.SP. Selection of two-mixture solvent**

To control the solubility as well as acidity, basicity and ionic strength of the solution, it was aimed to estimate the effects of two-mixture solvents as electrolytes such as some water-soluble organic solvents and water. For this purpose, initially acetone: H_2_O (80:20, V/V, 10.0 mL) was tested. The results were shown according to the CVs in Fig. 8SP during the analysis of 0.040 ± 0.001 mol L^-1^ Salen at a scan rate of 100 mV s^-1^ scan rate at high strong basicity during using KOH solution (0.01 mol L^-1^, 5.0 mL). As clearly evaluated (Fig. 8SP), the anodic peaks were observed only at the strong basic conditions. This effect clearly pointed to the negative charge of Salen during the electrosynthesis of the Salen-based polymer. In addition, effects of different mixtures of solvents such as CH_3_OH, C_2_H_5_OH, and acetone with water (80:20, V/V, 10.0 mL) were individually evaluated according to the CVs shown in Fig. 8SP. In agreement with the results, maximum sensitivity (anodic peak current) was measured during the use of acetone: water (80:20, V/V, 10.0 mL) with 100 mV s^-1^ scan rate. Fig. 8SP also showed the CVs of the Salen solution (0.040 ± 0.001 mol L^-1^) through the use of different ratios of acetone: water such as 80:20, 70:30, and 60:40 (V/V, 10.0 mL). According to the results (Fig. 8SP), the highest sensitivity was observed during the use of acetone: water with volume ratio of 80:20 (V/V, 10.0 mL) that was selected as an optimum solvent ratio.


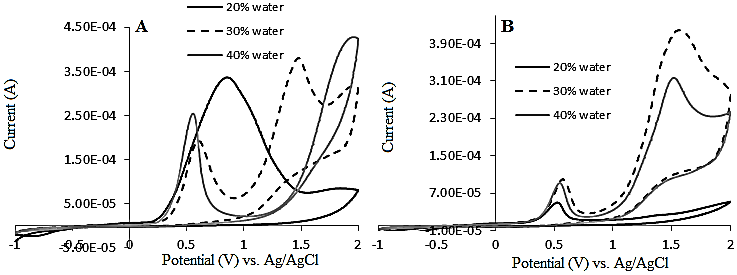


**Fig. 8 SP. |** Continuous cyclic voltammograms (CVs) of Salen-based monomer (0.040 ± 0.001 mol L-1), showing the effect of different ratios of acetonewater as two-mixture solvent at A) 1^st^ and B) 20^th^ scan cycles at potential ranging between -1.00 - +2.25 V (±0.01, vs. Ag/AgCl) and a scan rate of 100 mV s^-1^ at strong basic condition using KOH solution (0.01 mol L^-1^, 5.0 mL).

**16.SP. Effects of acidity/basicity and ionic strength**

The mixture of solvents such as acetone and water as an electrolyte, not only was miscible inside each other, but also provided the possibility to control the basicity and the ionic strength of the electrolyte. As demonstrated, no Salen-based polymer was generated under acidic conditions, due to the decomposition of Salen monomer.^2^ Therefore, optimization process was evaluated at different acidity and basicity conditions, partially with basic conditions. Fig. 9SP describes the CVs during the electrosynthesis using Salen solution inside KCl electrolyte with the same molar concentrations (0.040 ± 0.001 mol L^-1^) at 100 mV s^-1^ scan rate at different acidity and basicity values using different concentrations of KOH and HCl.


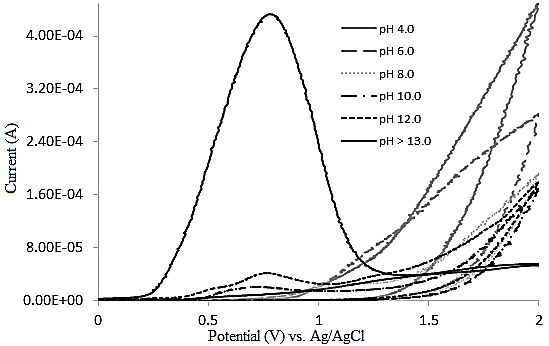


**Fig. 9 SP. |.** Continuous cyclic voltammograms (CVs) of Salen monomer (0.040 ±0.001 mol L^-1^) at different acid and basic conditions partally the same as pH values, ranged between 4 to >13 in the aqeous water medium using HCl and NaOH (0.01 mol L^-1^) inside acetone:water (80:20, V/V, 10.0 mL) at potential range between -1.00- +2.25 V (±0.01, vs. Ag/AgCl) and a scan rate of 100.0 mV s^-1^.

About the results (Fig. 9SP), the higher was the basicity, the more amount of Salen-based polymer was received. As a result, it was recommended to electro-synthesize the conjugated/unconjugated polymer at strong basic conditions such KOH solution. Compared to the acidic condition in which Salen was stable in the Salen H_2_ form, high basic conditions caused the salen in the form of Salen^2-^. This form not only promoted the solubility of Salen in the aqueous solution, but also accelerated the mass transfer rate. At this condition, high enough ionic strength was made for the electro-polymerization process. This was considered as another proof for the effect of basicity on the charge transfer during the anodic electro-polymerization process according to the recommended proposal behavior.

As the synthesized Salen-based polymer with negative charge had been tightly immobilized to the GC electrode, applying any negative potentials to the electrode system during the potential sweeping also led to the coordination of the cations such as K^+^, probably due to the complex affinity and electrical charge attractions.

**17.SP. Salen concentration**

Concentration of Salen monomer was also optimized to reach the maximum peak currents during the formation of conjugated/unconjugated Salen-based polymer. For this purpose, different concentrations of Salen ranged between 0.010 and 0.050 mol L^-1^ were tested. The results have been shown according to the voltammogram shown in Fig. 10SP during the use of acetone: H_2_O (80:20, V/V, 10.0 mL) as a high basic condition using KOH solution with 0.01 mol L^-1^ concentration (5.0 mL) and 0.040 ±0.001 molar concentration for KCl solution. Therefore, due to the maximum sensitivity of anodic peak current from 0.040 ± 0.001 mol L^-1^ concentration of Salen monomer, this concentration was selected as the optimum value for the electrosynthesis process.


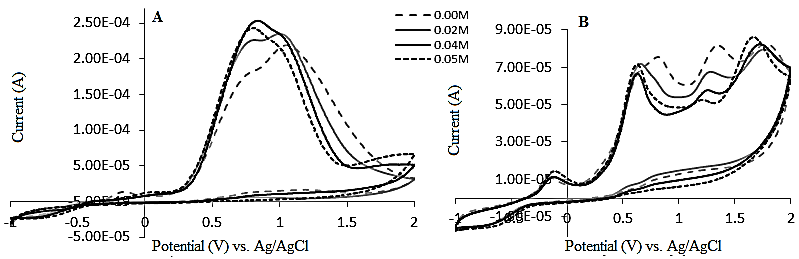


**Fig. 10 SP. |.** Continuous cyclic voltammograms (CVs) of Salen monomer (0.040 ±0.001 mol L^-1^) showing the effect of different concentrations of Salen monomer using acetone:water as two-mixture solvent at A) 1^st^ and B) 20^th^ scan cycles at potential ranging between -1.0 - +2.25 V (±0.01, vs. Ag/AgCl) and a scan rate of 100 mV s^-1^ at strong basic condition using KOH solution (0.01 mol L^-1^, 5.0 mL).

**18.SP. Optimum ratio of Salen/K^+^**

The UV-Vis. spectrophotometry was also adopted to estimate the ratio of K^+^/Salen. For this purpose, different mole ratios of K^+^/Salen monomer between 1:6 and 6:1 were prepared. The absorbance spectra were then measured as wavelength between 460-580 nm. According to the results, the optimum ratio of K^+^/Salen monomer in the coordination complex was estimated to be 5.35 ± 0.01 (n=3). The same correlation was evaluated during testing other alkaline ions such as Li^+^ and Na^+^. Estimation of this value was probably attributed to the different phenomena such as the orientation of the conjugated/unconjugated Salen-based polymer during the interaction with alkali ions such as K^+^.^7–11^. This result clearly revealed the coordination between the Salen-based polymer derivatives and alkaline ions such as K^+^.

This behavior of the synthesized polymer simply led to control the electrical charge of the electrosynthesized conjugated/unconjugated Salen-based polymer during the fabrication of supercapacitor. A strong interaction was therefore detected for the K^+^ during the interaction with the Salen-based derivatives. This result was also evidenced during analysis of free Salen and Salen/K^+^ with the FT-IR spectrometry. On the basis of the FT-IR spectra, strong absorption peak at around 3250 cm^-1^ was related to the hydration of Salen/K^+^ with H_2_O. Moreover, a significant shift from approximately 1550 to 1520 cm^-1^ was related to the absorption peak of C=N during interaction with K^+^. Thus, a strong interaction was detected between Salen and K^+^.


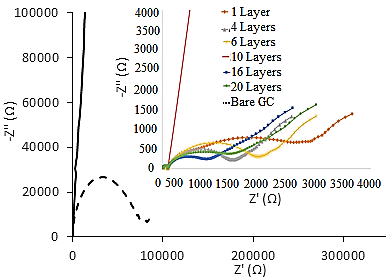


**Fig. 11 SP. |** Imaginary-real impedance (Nyquist) plots of GC and Salen-polymer-modified GC, inset) effect of different layers (self-assembled) of GC modified Salen-based polymer. Inset : Magnified Nyquist plots at different cyclic cycles. **Conditions**: Acetone :water as two-mixture solvent at potential ranging between -1.0 - +2.25 V (±0.01, vs. Ag/AgCl) and a scan rate of 100 mV s^-1^ at high strong basicity using KOH solution (0.01 mol L^-1^, 5.0 mL).


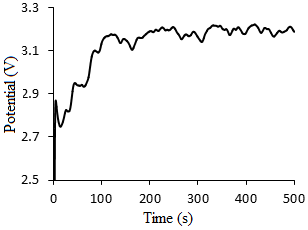


**Fig. 12 SP. |** Chrono-potentiogram (potential vs. time curve) of conjugated/unconjugated Salen-based polymer during applying potential at optimum condition. **Conditions**: Acetone :water as two-mixture solvent at potential ranging between -1.0 - +2.25 V (±0.01, vs. Ag/AgCl) and a scan rate of 100 mV s^-1^ at high strong basicity using KOH solution (0.01 mol L^-1^, 5.0 mL).

**Table 1 SP |** Estimation of electrical parameters of different layers of the polymer using Nyquist plot.

| **Electrode** | **R_s_** | **R_s_ + R_ct_** | **R_ct_** | **C_dl_** |
| --- | --- | --- | --- | --- |
| 1^st^ layer | 25 | 114 | 89 | 9.11×10^-9^ |
| 4^th^ layer | 28 | 109 | 81 | 1.00×10^-8^ |
| 6^th^ layer | 29 | 108 | 79 | 1.03×10^-8^ |
| **10^th^ layer** | **26** | **112** | **86** | **1.08**×**10^-8^** |
| 16^th^ layer | 25 | 115 | 90 | 1.05×10^-8^ |
| 20^th^ layer | 25 | 115 | 90 | 1.05×10^-8^ |
| **GCE** | **34** | **128** | **94** | **8.63**×**10^-9^** |
| **Conditions**: Acetone :water as two-mixture solvent at potential ranging between -1.0 - +2.25 V (±0.01, vs. Ag/AgCl) and a scan rate of 100 mV s^-1^ at high strong basicity using KOH solution (0.01 mol L^-1^, 5.0 mL). | | | | |

**Table 2 SP |.** Electrical parameters related to the 10^th^ layer of the conjugated/unconjugated Salen-based polymer using Nyquist plot.

| \| **Parameter** \| **Value** \| \| --- \| --- \| \| Moles of Used Electrons (mole ) \| 1.68×10^-8^ \| \| Mass of Synthesized Polymer ( g ) \| 1.13×10^-6^ \| \| Calculated Capacity ( F )= I dt/dV \| 1.00×10^-3^ \| \| Geometrical Surface Area ( cm^2^ ) \| 3.14×10^-2^ \| \| Capacity per Gram ( Fg^-1^ ) \| 888.30 \| \| Capacity per Volume ( F cm^-3^ ) \| 3.538.57 \| \| Volume (mL) \| 2.83×10^-7^ \| \| Density (g mL^-1^) \| 3.98 \| \| **Conditions**: Acetone :water as two-mixture solvent at potential ranging between -1.0 - +2.25 V (±0.01, vs. Ag/AgCl) and a scan rate of 100 mV s^-1^ at high strong basicity using KOH solution (0.01 mol L^-1^, 5.0 mL). \| \| |
| --- | --- | --- | --- | --- | --- | --- | --- | --- | --- | --- | --- | --- | --- | --- | --- | --- | --- | --- | --- | --- |

**References**

1. Cozzi, P. G. Metal–Salen Schiff base complexes in catalysis: practical aspects. Chem. Soc. Rev. **33**, 410–421 (2004).

2. Kingsborough, R. P., & Swager, T. M. Electroactivity enhancement by redox matching in cobalt salen–based conducting polymers. Adv. Mater. **10**, 1100–1104 (1999).

3. Simon, P., Gogotsi, Y. & Dunn, B. Where do batteries end and supercapacitors begin? Science (80-. ). **343**, 1210–1211 (2014).

4. Brousse, Th., B´elanger, D., & Long. J. W. To be or not to be pseudocapacitive?,. J. Electrochem. Soc **162**, A5185-A5189. (2015).

5. Blout, E. R., Fields, M. & Karplus, R. Absorption Spectra. VI. the infrared spectra of certain compounds containing conjugated double bonds. J. Am. Chem. Soc. **70**, 194–198 (1948).

6. Bühlmann, K. Umezawa, K. Tohda, S. A. Y., & Umezawa, Ph. Potentiometric selectivity coefficients of ion-selective electrodes. part I. inorganic cations,. Pure Appl. Chem. **72**, 1851 (2000).

7. Dadamos, T. R. L. & Teixeira, M. F. S. Electrochemical sensor for sulfite determination based on a nanostructured copper-salen film modified electrode. Electrochim. Acta **54**, 4552–4558 (2009).

8. Farias, P. A. M., & Bastos, M. B. R. Electrochemical behavior of copper(II) salen in aqueous phosphate buffer at the mercury electrode,. Int. J. Electrochem. Sci **4**, 458-470 (2009).

9. Goldsby, K. A., Blaho, J. K. & Hoferkamp, L. A. Oxidation of nickel(II) bis(salicylaldimine) complexes: Solvent control of the ultimate redox site. Polyhedron **8**, 113–115 (1989).

10. Li, M., Jiao, H., Zhang, H., & Jiao, Sh. Electrochemical polymerization of Schiff base transition metal polymer poly [Ni (Salen)] and its electrochemical performance in organic electrolyte,. Int. J. Electrochem. Sci. **10**, 8797-8806. (2015).

11. López, G. P., Castner, D. G., & Ratner, B. D. Xps O1s binding energies for polymers containing hydroxyl, ether, ketone and ester groups,. Surf. Interf. Anal. **17**, 267-272 (1991).
